# Activation robustness with directional leads and multi-lead configurations in deep brain stimulation

**DOI:** 10.1088/1741-2552/ab7b1d

**Published:** 2020-03-20

**Authors:** Andrew P Janson, Daria Nesterovich Anderson, Christopher R Butson

**Affiliations:** 1 Department of Biomedical Engineering, University of Utah, Salt Lake City, UT, United States of America; 2 Scientific Computing and Imaging (SCI) Institute, University of Utah, Salt Lake City, UT, United States of America; 3 Department of Neurosurgery, University of Utah, Salt Lake City, UT, United States of America; 4 Departments of Neurology, and Psychiatry, University of Utah, Salt Lake City, UT, United States of America

**Keywords:** neuromodulation, computational model, finite element, optimization

## Abstract

**Objective.:**

Clinical outcomes from deep brain stimulation (DBS) can be highly variable, and two critical factors underlying this variability are the location and type of stimulation. In this study we quantified how robustly DBS activates a target region when taking into account a range of different lead designs and realistic variations in placement. The objective of the study is to assess the likelihood of achieving target activation.

**Approach.:**

We performed finite element computational modeling and established a metric of performance robustness to evaluate the ability of directional and multi-lead configurations to activate target fiber pathways while taking into account location variability. A more robust lead configuration produces less variability in activation across all stimulation locations around the target.

**Main results.:**

Directional leads demonstrated higher overall performance robustness compared to axisymmetric leads, primarily 1–2 mm outside of the target. Multi-lead configurations demonstrated higher levels of robustness compared to any single lead due to distribution of electrodes in a broader region around the target.

**Significance.:**

Robustness measures can be used to evaluate the performance of existing DBS lead designs and aid in the development of novel lead designs to better accommodate known variability in lead location and orientation. This type of analysis may also be useful to understand how DBS clinical outcome variability is influenced by lead location among groups of patients.

## 1. Introduction

Deep brain stimulation (DBS) is an established therapy that delivers electrical stimulation to treat a growing number of neurological and psychiatric disorders. The efficacy of this surgical intervention was first demonstrated for essential tremor (Benabid *et al* 1996) and Parkinson’s disease (Obeso *et al* 2001), which has led to explorations of its use in other conditions such as treatment-resistant depression (Mayberg *et al* 2005), traumatic brain injury (Schiff *et al* 2007), and Tourette syndrome (Vandewalle *et al* 1999). Historically, the initial surgical targets for these disorders have been anatomical nuclei; however, evidence suggests that therapeutic effects are elicited through excitation of fiber pathways (Holsheimer *et al* 2000, Hashimoto *et al* 2003, Mcintyre *et al* 2004a). Recently, direct targeting of white matter pathways has been proposed for tremor (Schlaepfer *et al* 2013), depression (Riva-Posse *et al* 2014, Coenen *et al* 2018) and traumatic brain injury (Schiff 2012).

Although DBS therapy can provide substantial improvement in symptoms and quality of life, outcomes for patients can be highly variable. Standard deviations in primary outcome measures are often larger than the effect size, as shown in past clinical trials (Weaver *et al* 2009, 2012, Odekerken *et al* 2013). Two critical factors underlying this variability are the location and type of stimulation (Machado *et al* 2006, Volkmann *et al* 2006, Johnson *et al* 2019). Despite progress in imaging protocols and surgical techniques, DBS lead placement can exhibit considerable variability across patient cohorts, for which several possible reasons have been proposed. Brain structures can shift up to 4 mm when the cranium is opened during surgery (Winkler *et al* 2005, Khan *et al* 2008, Pallavaram *et al* 2010). Meta-analyses have reported that over 45% of implants are off-target in patients who experience suboptimal therapeutic effects (Okun *et al* 2005, Rolston *et al* 2016). Revisions to lead placement with a second surgery or better titration of stimulation parameters have been reported to improve clinical outcomes in these patients (Ellis *et al* 2008, Pourfar *et al* 2016).

Computational models of DBS have shown that minor variations in lead location and the shape of the stimulation field can influence the effects of activation on the target region (Butson and Mcintyre 2008, Chaturvedi *et al* 2012, Lehto *et al* 2017, Anderson *et al* 2018). These findings demonstrate that the ability to deliver stimulation to a target brain region is crucial to provide therapeutic benefit, and therefore we need to understand which lead designs and configurations are less sensitive to lead location variability.

Over the past few years, new directional DBS electrode geometries have been introduced, improving upon the existing axisymmetric cylindrical electrodes by providing the ability to steer stimulation around the lead. The implementation of multi-lead designs, i.e. multiple DBS leads close to one another, allows for the stimulation field to be shaped across leads over a broader region of brain tissue and has been demonstrated to elicit improved effects compared to single leads in both clinical (Oliveria *et al* 2017) and pre-clinical studies (Baker *et al* 2016). Both directional and multi-lead paradigms were developed to limit the negative impact of lead location variability and the resulting off-target side effects. The goal of these approaches is to provide a more robust therapy that is capable of providing therapeutic stimulation. However, no comprehensive study has evaluated how these novel DBS devices reduce stimulation variability and what measurable improvements they provide over the standard axisymmetric electrodes when considering the uncertainty of lead or target locations. This knowledge could guide the development of new electrode designs and targeting strategies to improve patient outcomes.

The objective of this study is to evaluate the ability of novel directional electrode designs and multi-lead configurations to robustly activate target fiber pathways to better understand how DBS technology can be improved to handle lead location variability. Our goal is to apply a robustness measure, defined as the ability to perform consistently across a known range of variability, to evaluate the performance of DBS lead designs and configurations. In the context of DBS, robustness is the ability to achieve activation of the target region despite variations in lead or target location. We characterize the performance of directional leads or multiple lead configurations compared to the current cylindrical electrode lead designs. This analysis is impractical to implement *in vivo* since lead location is fixed after surgery and can be changed only with subsequent revision surgeries. Therefore, we investigated a range of electrode positions in a computational model. These techniques allow direct comparison of performance variability across any existing DBS lead designs, including multi-lead configurations, as well as the capability to test novel electrode designs before they are manufactured.

## 2. Methods

We define a measure of stimulation robustness and use computational models to perform a quantitative evaluation of performance across several lead designs and configurations. The computational model includes a representation of neuronal axons as a stimulation target alongside virtual representations of various DBS electrode geometries. We evaluated the ability of the lead configurations to stimulate a target white matter fiber pathway across a representative range of lead placements.

### 2.1. Experimental model

#### 2.1.1. Finite element computational model

A finite element model (FEM) was created in SCIRUN 5.0 (SCI Institute, University of Utah, Salt Lake City, UT) to solve the Poisson equation and compute the voltage distribution in tissue surrounding active DBS electrodes. We used a previously published simulation pipeline that provides near real-time tetrahedral mesh generation and bioelectric field simulation (Janson and Butson 2018). This approach allowed us to place different electrode geometries in the same model and rapidly test new electrode configurations, including multi-lead designs with leads in proximity to one another. Each tetrahedral mesh generation and simulations required about 10–20 s, which enabled us to test many electrode placement locations around the target region and explore the effects of multi-lead spacing on neuronal stimulation.

**Figure 1.**
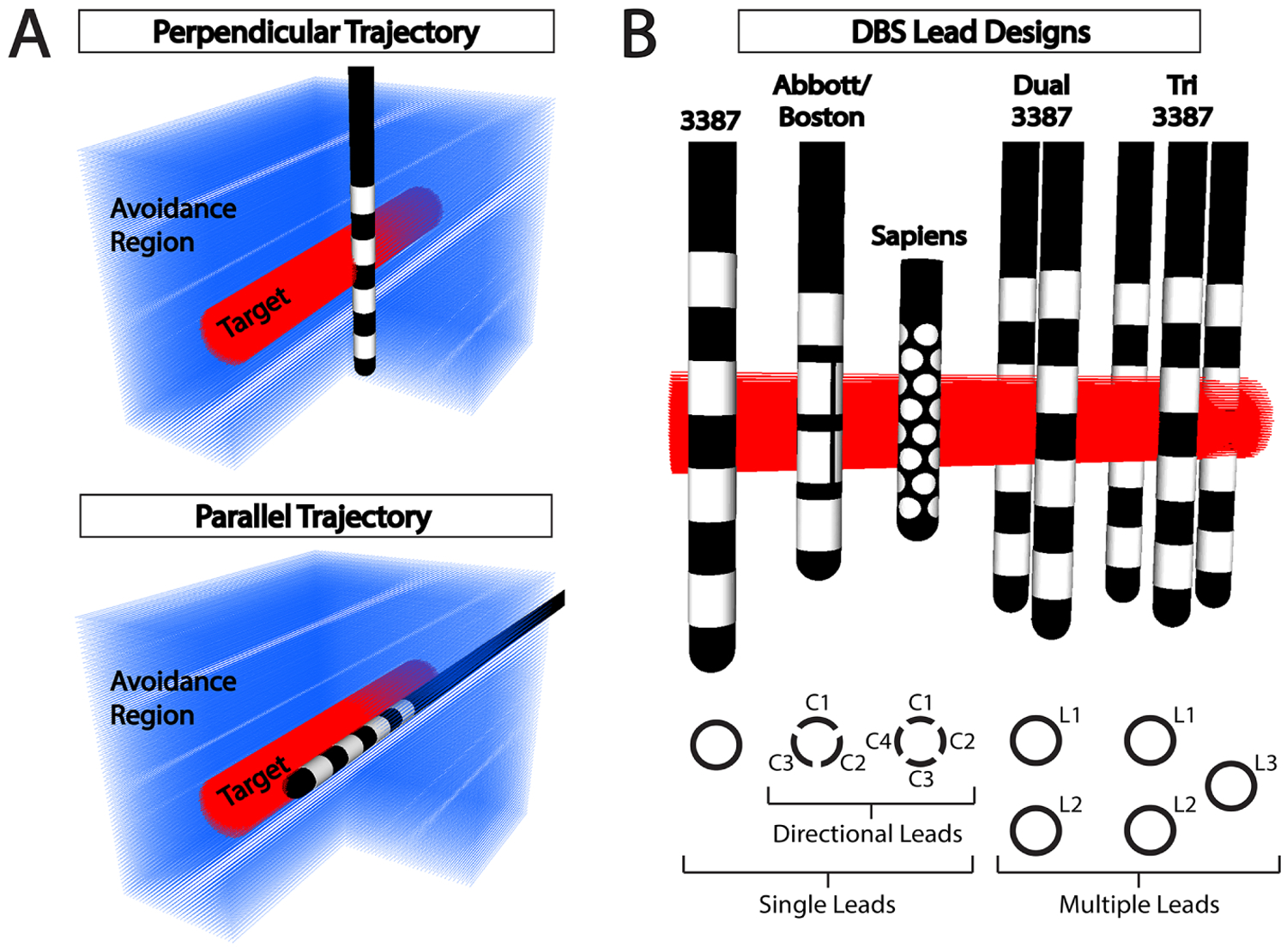
Simulation domain for robustness analysis and diagram of tested lead designs. (A) 3D visualization of a classic deep brain stimulation (DBS) lead next to the target fiber bundle (red) and fibers in the avoidance region (blue) for both a perpendicular and parallel approach to the target. (B) Diagram of the five tested lead designs in a perpendicular orientation to the target fiber bundle (red): (1) a single Medtronic 3387 lead, (2) a single Abbott or Boston Scientific directional lead, (3) a single Medtronic Sapiens directional lead, (4) two (Dual) Medtronic 3387 leads, and (5) three (Tri) Medtronic 3387 leads.

We defined the target region in the experimental setup as a 3 mm diameter cylindrical white matter fiber bundle with parallel axonal projections. Axons within the cylinder had internodal spacing of 0.5 mm with a total length of 20 mm and were evenly spaced 0.1 mm apart. The avoidance region, representing off-target stimulation, was defined using the same horizontal axonal projections with a uniform spacing of 0.25 mm increments outside the target cylinder and up to 8 mm away from the center of the target (figure 1(A)). Nodes from both the target and avoidance axons were included in the construction of the tetrahedral meshes to improve accuracy when solving for the electric potential along the axon. Any axon that intersected with the lead was removed from the simulation. All calculations for percent activation of the target bundle were normalized by the total number of axons. The outer boundary of the tetrahedral domain was a 100 × 100 × 100 mm cube.

A uniform 3D grid with 0.25 mm spacing up to 5 mm away from the center of the target was created to evaluate target activation across a realistic range of lead placements based on statistics from human DBS studies (Mcclelland *et al* 2005, Ellis *et al* 2008, Lee *et al* 2018). Each point in the grid defined a lead location to construct a new tetrahedral mesh and solve for the bioelectric field at each electrode. Both perpendicular and parallel electrode orientations were tested (figure 1(A)), representing two extremes of how DBS leads can approach a target fiber bundle.

The Poisson equation, with Dirichlet boundary conditions, was solved to calculate the extracellular electric potential (*V*_*e*_) at every node in the tetrahedral mesh. The bioelectric field forward problem was solved using a point source set to −1 V at the center of the active electrode and the outer surface of the simulation domain set to 0 V to represent a distant return electrode. Only cathodic voltage-controlled stimulation was used in this study. Isotropic conductivities were applied for the electrical electrodes at sigma = 1 × 10^6^ S m^−1^ (Miocinovic *et al* 2006), the shaft segments at *σ* = 1 × 10^−10^ S m^−1^, and the avoidance region brain tissue at *σ* = 0.2 S m^−1^. Anisotropic conductivity tensors were applied to the target fiber bundle region at a ratio of 9:1, 0.9 S m^−1^ in the longitudinal direction and 0.1 S m^−1^ in the transverse direction of the fibers (Nicholson 1965). Although currently available implantable pulse generators are not capable of supporting independent voltage sources, we chose this approach to provide a consistent stimulation paradigm across all lead designs and configurations.

#### 2.1.2. DBS lead designs

Three classifications of lead designs, or configurations, were modeled in this study: (1) axisymmetric leads such as the Medtronic 3387 or 3389, (2) directional leads such as the Abbott Infinity, Boston Scientific, and Medtronic Sapiens, and (3) multi-lead designs with two or three axisymmetric leads close to one another. The Medtronic 3387 consists of four electrical contacts along a cylindrical shaft, each 1.5 mm in height and 1.27 mm in diameter with 1.5 mm spacing between each electrode. The Abbott Infinity and Boston Scientific directional leads have electrode geometries similar to that of the Medtronic 3387, except the two middle electrodes are split into three directional electrodes instead of one axisymmetric cylindrical electrode. Only the Abbott 6172 directional lead was modeled in this study because the geometric differences are minimal compared to the Boston Scientific lead. The Medtronic Sapiens directional lead uses rows of four circular electrodes at 90° increments around the lead. The multi-electrode designs were modeled with two and three Medtronic 3387 leads close to one another. The distance between two leads is measured from center to center and the tri-lead design is created from the dual-lead design by placing a third lead to create an equilateral triangle. The distance between the leads was determined by identifying the spacing at which the two leads were no longer able to maximally activate the target despite being centered on the target. This spacing remained fixed for all subsequent simulations.

### 2.2. Simulation and analysis

#### 2.2.1. Simulation overview

Several steps were taken to generate a performance metric that could be used to evaluate the overall robustness of each lead design. The steps to evaluate one lead design are summarized below and each step is explained in greater detail throughout the methods sections.

- Generate new finite element mesh (FEM) for a given lead location (section 2.1.1).
- Solve the bioelectric field for each electrode in the lead during monopolar stimulation (section 2.1.1).
- Run the optimization algorithm to determine stimulation amplitudes (section 2.2.2).
- Calculate target activation and performance metric values (section 2.2.3).
- Move the lead to a new location and repeat the process.

**Figure 2.**
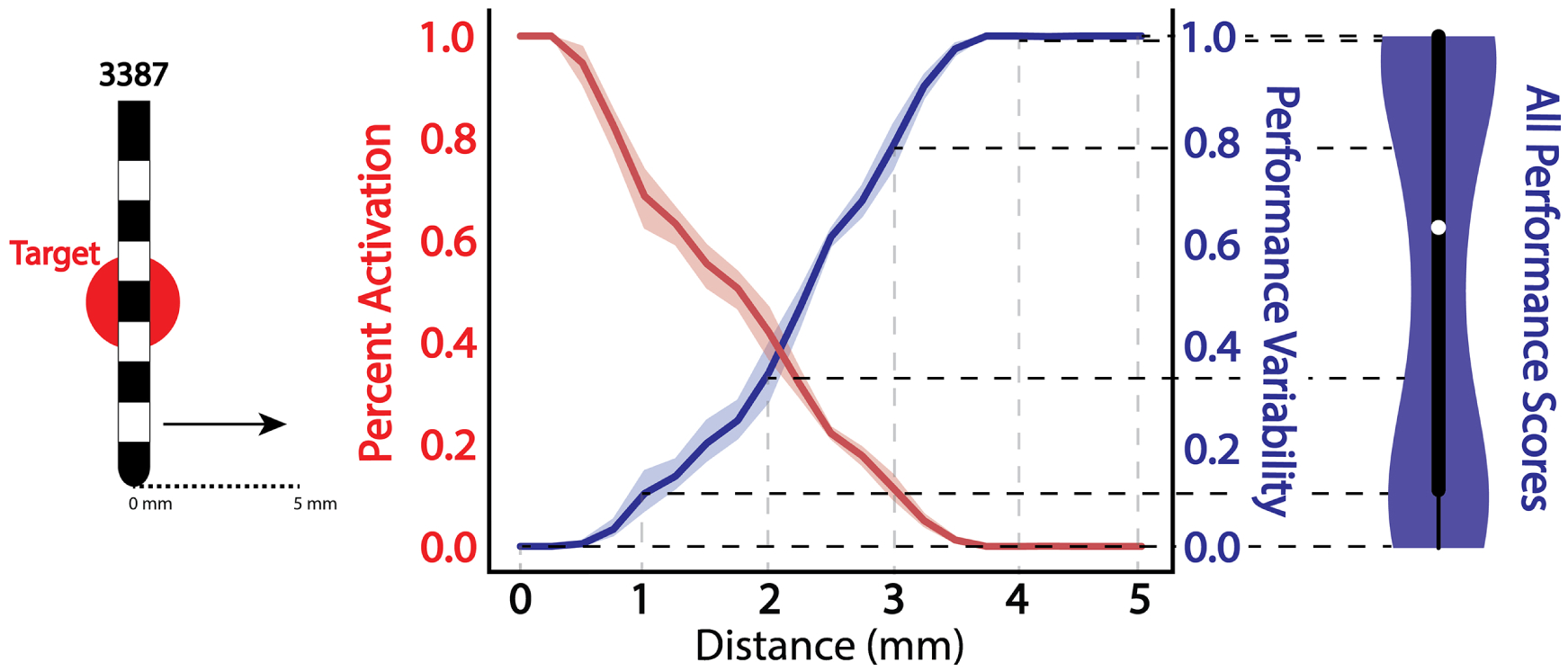
Performance variability evaluation across a single lead configuration. Left: a cross-section of a ssssingle Medtronic 3387 lead and target fiber bundle with the corresponding percentage of fiber bundle activation (red) and performance variability calculation (blue) as a function of distance from the target. The shaded regions represent variance resulting from changes in the vertical depth (up/down movement) of the lead. Right: the corresponding violin plot and box plot of overall performance variability with a perpendicular trajectory for the single lead design that encompasses variability scores across all simulated lead locations. Overall performance throughout the entire simulation space is depicted with both a box plot to show median performance (white circle) with quartiles and a violin plot to depict a continuous distribution of performance scores.

Figure 2 demonstrates how each step contributes to an overall robustness evaluation by averaging the performance metric across all tested lead locations for a single Medtronic 3387 lead. The figure illustrates both the percent fiber activation and resultant performance variation calculation as the lead moves over a range of representative positions. The violin plot for the 3387 lead illustrates that although the normalized performance metric has both a median and mean in the middle of the range, most of the data values are skewed toward the maximum and minimum of the scale which is not evident from a box plot alone.

#### 2.2.2. Stimulation optimization

To compare the performance of lead designs with respect to variable lead positions around the target, we computed multiple bioelectric field solutions at each location on the 3D grid and determined voltage amplitudes for each electrode that maximally activated the target. We adapted a previously published method of automated DBS programming to provide a fast and objective way to optimize stimulation amplitudes for any lead design at any location in space around our target region (Anderson *et al* 2018). The optimization algorithm works by individually adjusting the voltage amplitude at each electrode to provide maximal stimulation to the defined target region while minimizing stimulation to any specified areas of avoidance. We calculated n number of extracellular potential solutions for each lead, where n is the number of electrodes in the lead configuration (equation (1)). We utilized the superposition principle for optimized configurations to include multiple active electrodes. The voltage solution for each electrode was first multiplied by its optimized amplitude and then each electrode solution was linearly combined to compute the overall voltage solution generated by the optimized configuration. The activating function (AF), the second spatial derivative of extracellular potentials (V_e_), was then calculated at each node x along each axonal projection from its neighboring nodes x − 1 and x + 1 with an internode spacing of Δx (equation (2)). Neuronal activation, as a result of extracellular electrical stimulation, can be approximated by thresholding the activating function (Rattay 1986, 1999). As previously reported (Anderson *et al* 2018), the threshold values of the AF were established by comparison to multi-compartment cable model simulations and set to 15 mV, which is within the range of 5–30 mV established in other studies (Rattay 1986, Mcintyre *et al* 2004b, Martens *et al* 2011).

The *AF* calculated along each axonal projection in both the target and avoidance regions served as the input to the optimization algorithm, utilizing the CVX package in MATLAB for solving linear convex optimization problems (Grant and Boyd 2008). The maximum *AF* value found along each axonal projection is then extracted to provide a single value to represent the excitability of each axon in response to stimulation for each electrode in the lead configuration. This algorithm maximizes the objective function (equation (3)), which optimizes the cathodic voltage amplitude at each electrical contact (*c*_*i*_) that maximizes the average AF value for each axon (*AF*_*i,Target*_) in the target region (**Ω**_*Target*_), while minimizing the squared average *AF* value for each axon (*AF*_*i,Avoid*_) in the avoidance region (**Ω**_*Avoid*_). A penalty weighting of *s* for the avoidance region was set to a value of 8 for all simulations. The variable s is a free parameter in the optimization algorithm and will vary based upon the shape and size of the avoidance region. Adjustments to s will affect stimulation amplitudes but active electrode configurations will be conserved (Anderson *et al* 2018). The other constraint implemented in the optimization algorithm was a maximum charge density of 30 *μ*C cm^−2^ (Mccreery *et al* 1990, Shannon 1992) for each electrode design. After the stimulation amplitudes were optimized, we thresholded the *AF* along each axon to determine the total number of activated axons in the target region and the percentage of total fiber bundle activation for the given lead position. Avoidance region activation was implemented as a constraint in the objective function of optimization algorithm and therefore was not directly included in the performance metrics.

(1) $$\nabla \cdot \sigma \nabla V_{e}=-I$$

(2) $$A.F.=\frac{V_{e\left(x-1\right)}-2V_{e\left(x\right)}+\;V_{e\left(x+1\right)}}{\Delta x^{2}}$$

(3) $$f_{\nu ,\Omega \left(x,c\right)}=\sum_{n}^{i=1}\frac{c_{i}}{\left|\Omega_{\text{Target}}\right|}\text{max}\left(AF_{i,\text{Target}}\left(x\right)\right)-s\frac{c_{i}}{\left|\Omega_{\text{Avoid}}\right|}\text{max}{\left(AF_{i,\text{Avoid}}\left(x\right)\right)}^{2}$$

#### 2.2.3. Robustness metric analysis

We defined maximal performance as the ability to achieve total activation of the target fiber bundle, and our parameter variation is the lead location around the target. Realistically, each lead design or configuration should be able to provide maximal (100%) activation during ideal placement, i.e. when the lead is placed directly on target. Our goal was to quantify variations in performance as a function of lead location relative to the target. Therefore, the robustness metric (*R*), which is a measure of performance variability (*P*_*v*_) over the entire simulation domain, was calculated as a quadratic loss function (equation (4)) and averaged across all lead locations (equation (5)). The quadratic loss function penalizes larger drops in percent activation more than minor deviations. In DBS therapy applications, it is unknown whether 100% activation of the target is necessary to evoke a therapeutic response; however, the robustness metric characterizes the ability to do so, and admits a wide range of other penalty functions. Decreases in percent activation at a given location *(A*_*l*_) are predominantly controlled by the optimization algorithm limiting the stimulation of the avoidance region as the lead is placed farther off-target. Although the percent activation calculation is influenced by several parameter choices in the optimization algorithm, all lead designs were exposed to the same constraints to allow for the relative comparisons between them to be consistent. A lead design is determined to be more robust if the R metric is higher than other lead designs, meaning it is more likely to achieve target activation across the range of simulated lead locations.

(4) $$P_{v}\left(l,C\right)={\left(1-A_{l,C}\right)}^{2}$$

(5) R(C)=1−1L∑l=1LPv(l,C)

A_l_ = %Activation per location

C = Lead configuration

## 3. Results

### 3.1. Single lead target activation robustness

Robustness analysis was first investigated for the axisymmetric Medtronic 3387 lead. We observed that the 3387 lead was able to achieve maximal activation of the target fiber bundle when positioned at the center of the target. However, activation performance quickly dropped as the lead was moved off target and was effectively zero once the lead reached a distance of 3.75 mm.

**Figure 3.**
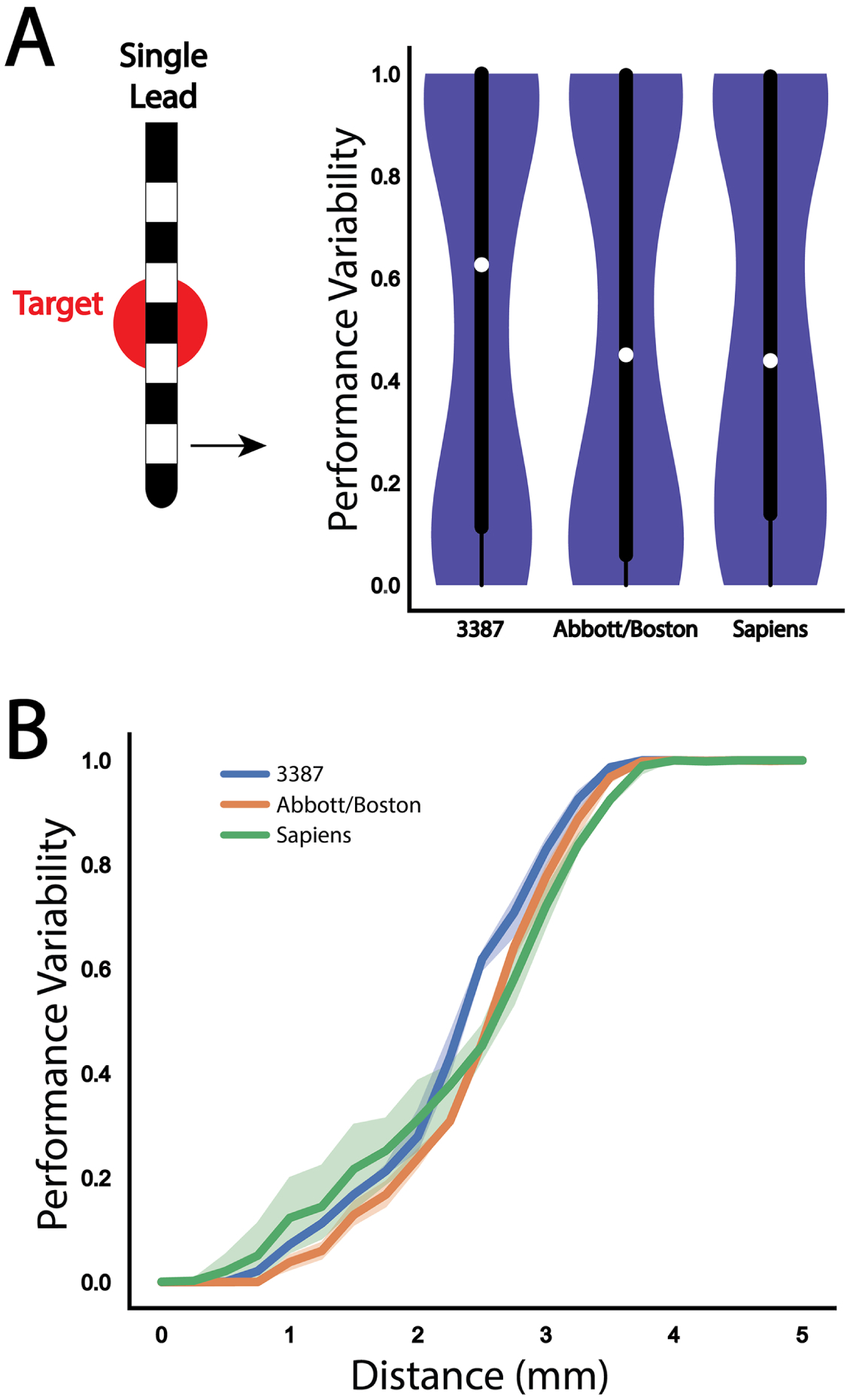
Performance variability evaluation across each single lead configuration. (A) Violin plot and box plot of overall performance variability for a perpendicular trajectory for each of the three single lead designs. (B) Comparison of performance variability as a function of distance from target for each of the three single lead designs: Medtronic 3387 (blue), Abbott 6172 (orange), Medtronic Sapiens (green). The shaded regions represent variance resulting from changes in the vertical depth (up/down movement) of the lead.

We repeated the analysis with the Abbott and Medtronic Sapiens directional leads (figure 3(B)). The Abbott lead provided better performance than the 3387 lead at all distances until both configurations failed to provide any activation of the target fiber bundle. Although the Medtronic Sapiens lead experienced a drop in performance earlier than the other two leads, the slope of decay was lower and eventually outperformed the Abbott lead at more distant off-target locations. Both of the directional leads were not able to produce any target activation at a distance of 3.75 mm, similar to the Medtronic 3387 lead. For all distances beyond 2.5 mm, all three lead designs demonstrated a similar decay in performance. The overall performance across all simulated lead locations for the three single lead designs is summarized in violin plots in figure 3(A), with the median performance and interquartile range (IQR) of the Medtronic 3387, Abbott, and Medtronic Sapiens leads found to be 0.63 (IQR = 0.89), 0.45 (IQR = 0.94), and 0.44 (IQR = 0.86), respectively. The corresponding robustness metrics for the three leads were: 0.46, 0.49, and 0.48, respectively.

### 3.2. Multi-lead optimal spacing

**Figure 4.**
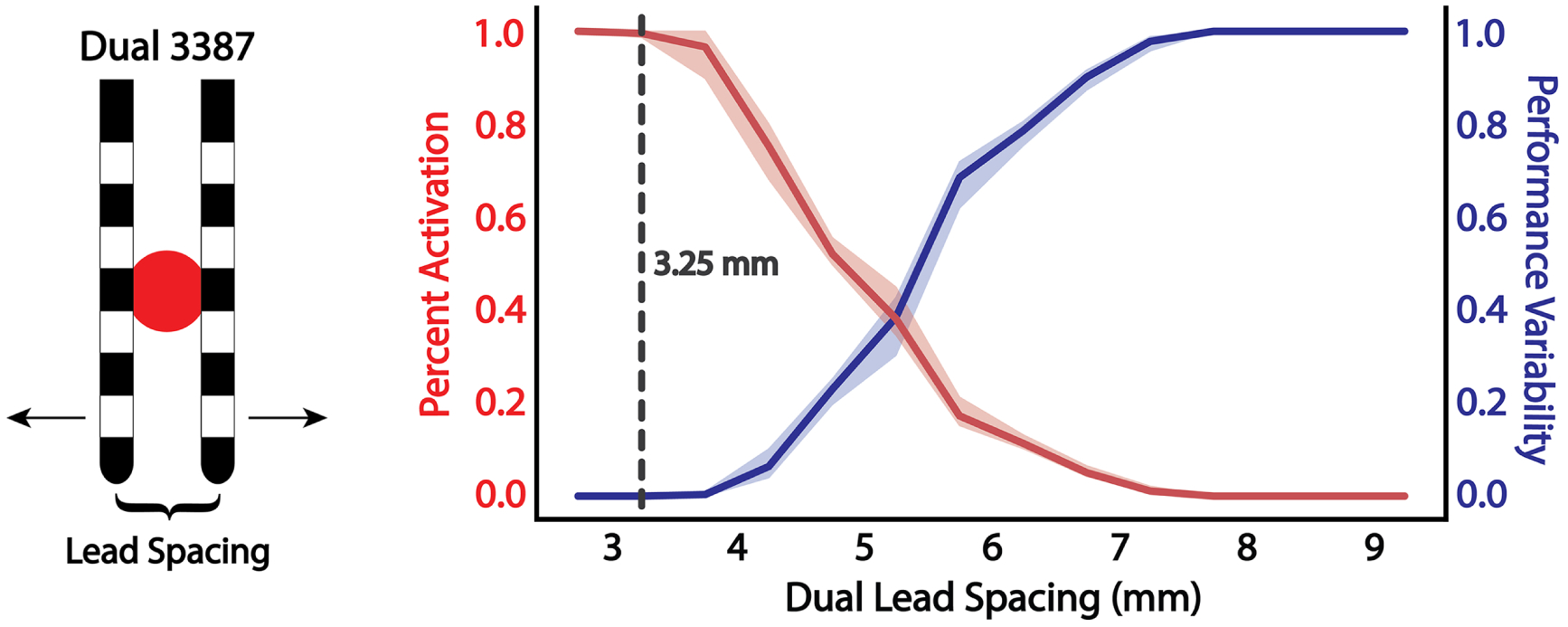
Evaluation of optimal dual lead spacing to provide maximum stimulation coverage of the target region. Percent fiber bundle activation (red) and performance variability (blue) as a function of inter-lead spacing with a cut-off at 3.25 mm to be used in subsequent robustness analyses. The shaded regions represent variance resulting from changes in the vertical depth (up/down movement) of the lead.

The goal of using multiple leads in proximity is to move each of them away from the direct center of the target to provide stimulation over a broader region while maintaining the ability to activate the target maximally. However, the spacing between the leads cannot be so far apart that they are no longer able to activate the center of the target. A diagram of the multi-lead configuration with two Medtronic 3387 leads is shown in figure 4. The midpoint of the leads determines the distance to target for multi-lead configurations.

The interlead spacing was held constant during the robustness analysis to reduce the parameter space for multi-lead configurations. Therefore, we need to determine the optimal lead spacing that can provide stimulation to the largest region possible while also maintaining the ability to activate fibers at the midpoint of the two leads. For this experiment, the midpoint of the two leads was held constant at the center of the target fiber bundle, and the two leads then moved horizontally away from one another in 0.25 mm increments. The percentage of fiber activation and resulting robustness metric was calculated for each interlead spacing distance, shown in figure 4(B). The optimal interlead spacing was identified by the distance just before maximal activation of the target started to decline. If the leads were separated any farther apart, maximal activation of the target would not be possible under the ideal scenario, which is the midpoint of the leads positioned directly on the target. The optimal spacing was determined to be 3.25 mm center-to-center spacing of the two 3387 leads. With this multi-lead configuration, each lead is positioned 1.625 mm off-target; however, stimulation through the combination of both leads is still able to provide maximal activation of the target fiber bundle. As a reference from our previous result, a single Medtronic 3387 lead positioned 1.625 mm off-target was able to provide approximately 50% activation.

### 3.3. Multi-lead rotational dependence

**Figure 5.**
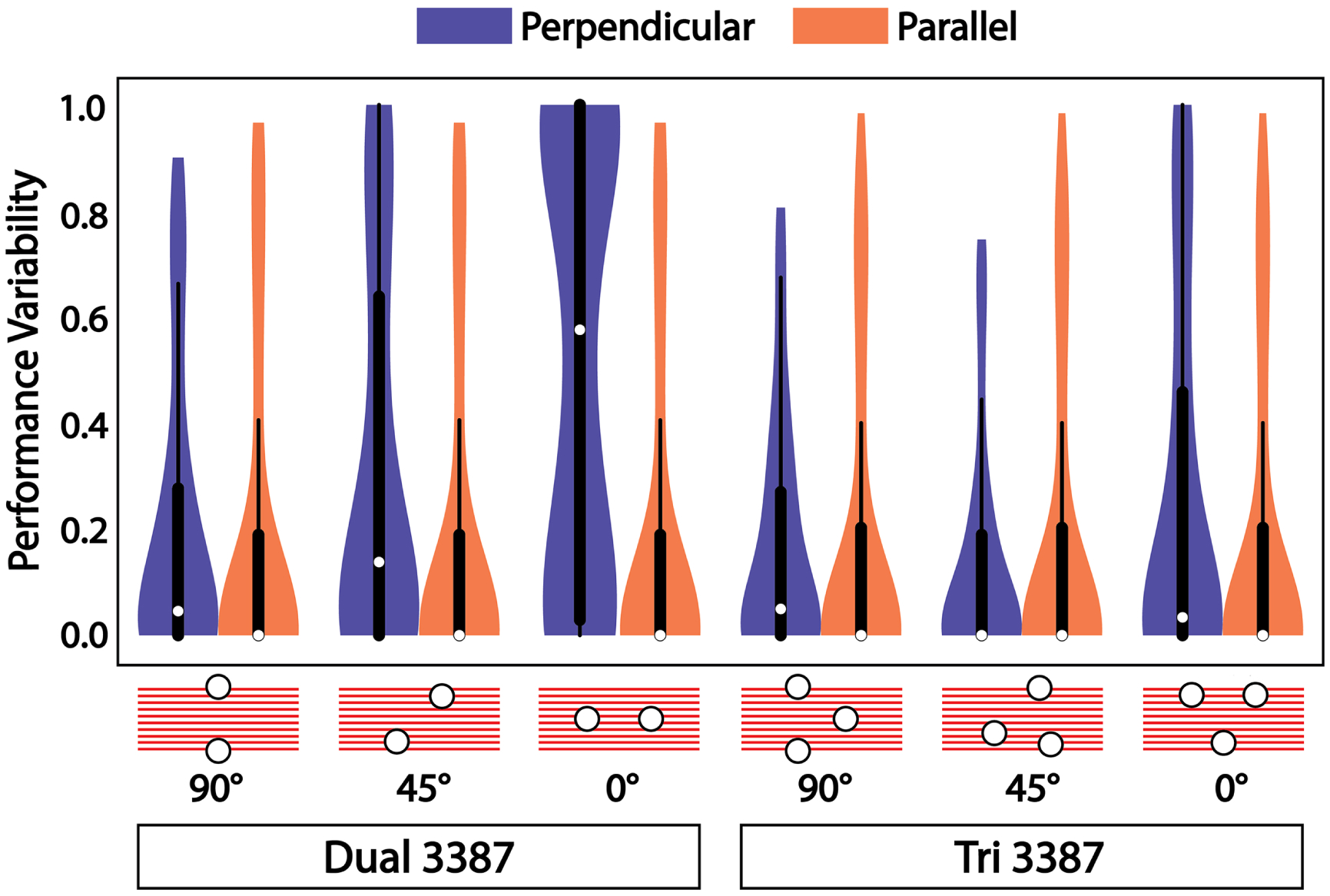
Dependence of multi-lead configurations on rotational orientation with respect to the target. Violin and box plots of overall performance variability across the simulation domain for perpendicular (blue) and parallel (orange) approaches to the target with multi-lead configurations. The dual Medtronic 3387 performance is dependent on rotational orientation with respect to the target for perpendicular approaches to the target producing better performance with leads oriented across the target fiber bundle versus leads oriented along the same direction as the target fiber bundle, while the tri 3387 configuration demonstrates no major dependence on orientation.

The use of two leads, each offset from the target, introduces an asymmetry to the lead configuration that is dependent on its orientation relative to the fiber bundle. For a perpendicular approach to the fiber bundle, the leads themselves can be oriented perpendicular (90°), parallel (0°), or somewhere in between, as shown in figure 5. We found that for a perpendicular approach to the target (blue violin plots in figure 5), the orientation of the two leads had a drastic effect on the robustness of the system and its ability to activate the target over the range of all stimulation locations. An orientation of 90° produced the best performance, median = 0.08, whereas an orientation of 0° performed marginally better than a single Medtronic 3387 lead, median = 0.63. This asymmetry does not exist for parallel approaches to the target fiber bundle (orange violin plots in figure 5), which showed no difference as a function of orientation and outperformed all perpendicular approaches, median = 0.01.

We then implemented a multi-lead configuration of three leads with 3.25 mm equilateral spacing in an attempt to eliminate this observed rotational dependence. The performance of this electrode configuration was tested under the same rotational conditions for both perpendicular and parallel approaches, as was done with the dual-lead configuration. We found that the tri-lead configuration performed the same, median = 0.08, as the dual-lead at an orientation of 90°. The performance of the tri-lead configuration improved or remained the same for 45° and 0° orientations, demonstrating that three leads are sufficient to eliminate the rotational dependence of orientation relative to the target pathway. Again, there was no rotational dependence, and overall robustness performance increased for parallel approaches to the target.

### 3.4. Overall robustness comparison

**Figure 6.**
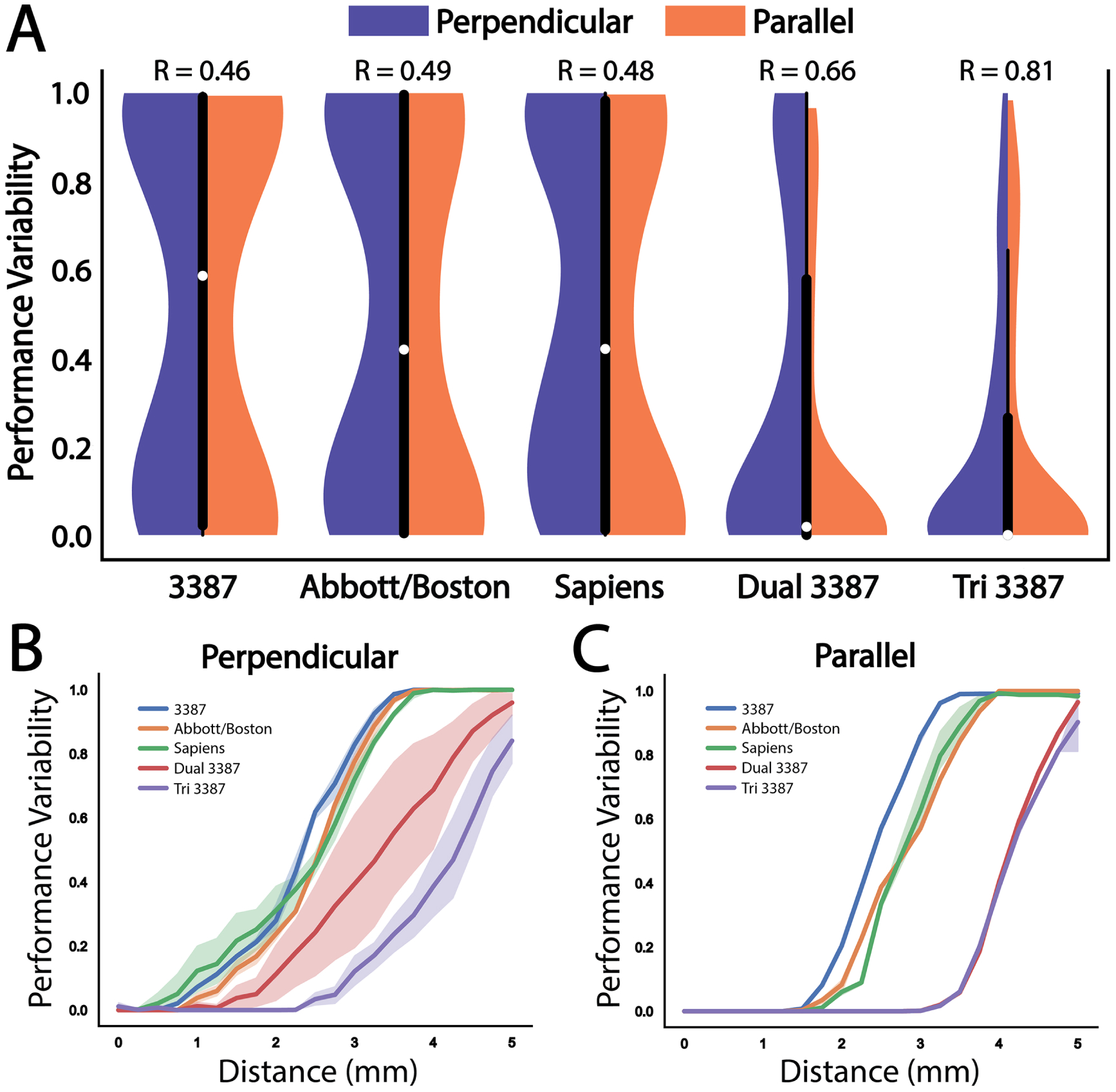
Performance variability for all lead configurations. (A) Violin plots for perpendicular (blue) and parallel (orange) trajectories with box plots of both trajectories combined for each of the five tested lead configurations. Each of the directional leads demonstrated performance improvements (lower variability scores) compared to the non-directional Medtronic 3387 lead. The multi-lead designs demonstrate markedly better performance over the directional leads. (B) Performance variability as a function of distance with a perpendicular angle of approach to target for each of the five lead configurations shows the greatest variability for the dual-lead configuration. The shaded regions represent variance resulting from changes in the vertical depth (up/down movement) of the lead. (C) Performance variability as a function of distance with a parallel angle of approach to target for each of the five lead configurations. In (B) and (C), the multi-lead configurations maintain performance robustness farther than any of the single lead configurations.

All possible locations and orientations relative to the target were combined for each lead configuration and are summarized in figure 6(A), with violin plots for perpendicular (blue) and parallel (orange) lead orientations relative to the target axons. A clear stratification emerges among the three configuration paradigms (axisymmetric, directional, multi-lead), with each increasing overall performance. The robustness metrics for the dual- and tri-lead configurations were 0.66 and 0.81, respectively. The tri-lead configuration produced an overall median value of 0 (IQR = 0.27), meaning it was able to provide maximal activation of the target in over half of the tested lead locations. The tri-lead configuration also produced minimal differences in overall performance between perpendicular and parallel approaches to the target compared to the dual-lead configuration, which, from the previous result, depended on orientation for perpendicular approaches.

The performance of each electrode configuration as a function of distance from the target for perpendicular and parallel lead orientations is shown in figures 6(B) and (C), respectively. These panels show how the two multi-lead configurations compare to the single lead configurations. Both multi-lead configurations maintain higher levels of performance over all distances, and the tri-lead configuration does not show a considerable drop in performance until 2.5 mm off target with a perpendicular approach and approximately 3.5 mm with a parallel approach. Both directional leads demonstrate clear separation in performance compared to the Medtronic 3387 when the leads are located entirely outside of the target region beginning 1.5 mm from center. The dual lead configuration shows high variance in the perpendicular approach due to orientation dependence, but the performance is nearly identical to the tri-lead configuration when the leads are parallel to the target.

## 4. Discussion

Despite the success of DBS as a therapy to treat numerous neurological disorders, not all patients receive the same benefit, in part, due to variable lead locations. New methods of objectively evaluating lead designs and targeting schemes are needed to guide future device designs that are less sensitive to this variability. The goal of this computational study was to establish the concept of robustness to compare the ability of modern DBS lead designs to activate therapeutic targets and investigate potential new configurations that can perform well across a range of locations around the target. We found that directional and multi-lead designs provide more robust control over activation of fiber pathways when accounting for variability in lead placement than cylindrical leads, and that multi-lead designs were more effective at robust target activation than directional leads.

We chose the metric of robustness to objectively evaluate the performance of one lead design versus another over a range of possible lead locations that could feasibly occur across a patient cohort. The rationale behind the design of the new directional leads was to reduce off-target, stimulation-induced side effects, but a quantitative evaluation of those lead designs, as reported in this manuscript, was not done before clinical testing. Our approach may be able to guide future lead designs during development and prior to adoption. Other robustness metrics may be useful during this process, such as the inclusion of power consumption or variable weighting of one or more avoidance region. Our current analysis process could include these constraints inside of the optimization algorithm, but it is also possible to optimize stimulation settings for target activation and then add these variables into the performance calculations which will allow us to analyze how much stimulation spreads into these avoidance regions.

Our model constrained the stimulation amplitude to limit the spread of activation to regions outside the target. The use of the optimization algorithm developed in Anderson *et al* (2018) provided an objective way to compute the optimal stimulation amplitudes across every simulated lead location and each lead design. Establishing the performance robustness metric allowed us to directly compare current designs as well as develop novel lead placement strategies that may provide more reliable therapeutic stimulation.

We summarized the performance of a single Medtronic lead across a range of lead locations since minimal computational research has been done to comprehensively quantify target activation as a function of lead placement (Chaturvedi *et al* 2012, Keane *et al* 2012, Hartmann *et al* 2015). Most previous studies that have explored any form of performance versus lead placement location have tested only a few, sometimes only two, lead locations due to computational limitations.

The performance benefits of the two directional lead designs were explored under the same conditions as the Medtronic 3387. We found no major differences in the performance of the two directional leads, but both performed better than the Medtronic 3387 lead. The difference in performance might not be as considerable as expected because our avoidance of off-target effects might be too punishing. Directional leads are thought to show their greatest efficacy when less than 1 mm outside the target, which is also where our results show the greatest separation between directional and axisymmetric leads. In some clinical cases, there might be a gap between target and off-target sites that does not produce any harm, which could produce a slightly greater functional distance at which these directional electrodes can activate the target. We also observe little variability with regards to depth placement compared to lateral placement, as long as the target falls within the span of the electrodes on the lead. Given that electrodes span 8–10 mm along the lead shaft, with minimal gaps in-between, we expected minimal performance variation with shifts in the vertical lead position. Further testing is needed to explore how the spacing between electrodes on a single lead would affect activation performance if the target falls between two electrodes, especially since the directional leads studied have much smaller electrode spacing that the Medtronic 3387 lead. We also found that rotating the directional leads about their axis had no significant impact on performance variability (data not shown). Pena *et al* (2018) reported similar findings in their study of optimization algorithms for directional leads. With a constant configuration of active electrodes they observed less than 10% variation in activation across a 360° rotation of the lead about its axis. We allowed for the configuration to change as the lead rotated, which most likely contributes to why we observed even less variation in activation.

Although the directional leads demonstrated increased performance compared to axisymmetric leads, the main limitation to activating nearby targets is that the stimulation field can be shaped around only a single lead. All the single lead designs showed similar decreases in performance as a function of distance from the target and similar maximum distances at which they were no longer able to activate the target. Therefore, we explored the use of multiple leads in proximity that enabled independent sources across leads. The electric potential can be shaped across a broader tissue region and can provide more selective activation within this region. In the simplest cases, much of the multi-lead performance can be attributed to increasing the odds that a lead is closer to the target compared to single lead implantations. Although this concept may be self-evident, the added dimension of placing additional electrodes around the target rather than segmenting a single lead has not been thoroughly explored. Extending the field of stimulation could be enough to reduce variability when factoring in the uncertainty of target identification and lead placement. The use of multiple leads has been demonstrated in a human clinical case (Oliveria *et al* 2017) that explored expanding the stimulation field to target both the ventralis intermedius (VIM) and the ventralis oralis (VO) nuclei in the thalamus for the treatment of multiple sclerosis tremor. Multiple leads have also been used in non-human primate research (Baker *et al* 2016) to target the central lateral nucleus and passing fibers in the central thalamus to control arousal regulation. The non-human primate stimulation found that bipolar shaping of the stimulation field across the two leads was more effective than any single lead stimulation which our study did not explore. Further studies are needed to compare the steering capabilities of directional versus multi-lead designs.

For perpendicular lead orientations relative to the target, with the dual-lead configuration, a rotational asymmetry quickly becomes apparent. Our simulations showed a strong performance dependency on rotational orientation with respect to the target. Placing two leads along the same direction as the target did not perform better than a single lead. However, enabling the use of bipolar configurations in the optimization algorithm might reduce the effect of rotational asymmetry for the dual-lead configuration. Multiple leads positioned across the target, demonstrated an increase in performance compared to any of the single lead configurations. A third lead was added to form an equilateral triangle under the assumption that this configuration would reduce the geometric asymmetry and enhance performance. The tri-lead configuration, although not wholly eliminating rotational dependence, drastically improved performance across all other orientations compared to the dual-lead configuration. For parallel approaches to the target, the dual-lead configuration performs as well as the tri-lead configuration, because no asymmetry exists. The improved performance of the multi-lead configurations can be mostly accounted for by always having a lead closer to the target than the single leads.

Combining simulation results for both approach angles and all possible orientations allowed us to compare the overall performance across each electrode configuration. Each increase in electrode configuration complexity, from single lead to directional lead to multiple leads, produced considerable increases in target activation performance. Directional leads were able to provide more robust activation of the target at a wider range of locations because they could steer current to one side of the lead and avoid most of the off-target region compared to the axisymmetric lead. Multi-lead designs were more robust than directional leads due to their ability to control stimulation over a broader spatial region, making their lead placement much less sensitive to off-target placements. In the best case scenarios, multi-lead designs maintained high levels of performance up to 3 mm off target, whereas all the single lead designs could not maintain performance past 1.5 mm and then exhibited a steeper decline in performance at greater distances.

A direct extension of this study would be to perform a robustness analysis for a specific clinical target that includes an accurate representation of the target itself and surrounding avoidance regions. Retrospective lead location data from previous patients could then be used to evaluate activation with realistic positions around the target instead of the uniform spacing used in this study. This study outlines an approach to evaluate how DBS lead choice affects the activation of both the therapeutic target and nearby regions of avoidance while accounting for plausible lead placements. This analysis could guide patient-specific clinical decisions about lead designs and trajectories to provide the greatest therapeutic benefit. The results generated in this study provide a baseline intuition about how different lead geometries activate a generic fiber pathway across a range of probable lead locations. We expect this analysis to produce different results for each specific clinical target based on its exact geometry and the surrounding side-effect regions.

### 4.1. Limitations

All computational models require several assumptions about the generation of the bioelectric field solution from the DBS lead and how to quantify neuronal activation. First, the activation predictions from thresholding the *AF* used in this study considered only the amplitude of the stimulation waveform, not pulse width, frequency, or overall shape of the waveform. Also, we explored only cathodic stimulation when optimizing electrode configurations that maximize activation of the target region. Thresholding the *AF* eliminated the computational bottleneck of running NEURON models and allowed us to simulate thousands of lead locations over five lead designs and configurations. The automated programming algorithm used in this study, developed by Anderson *et al* (2018), uses the *AF* to optimize stimulation amplitudes and validated thresholds against multi-compartment NEURON models. Second, all axons in both the target and off-target regions were modeled as parallel axons, which other computational studies have shown is the most excitable subunit of a neuron (Mcintyre and Grill 1999). We used a generic fiber tract target with an approximate size to compare the relative differences of fiber activation across lead designs and variable positioning, but the specific relationship of activation with respect to fiber bundle diameter was not investigated. Overall, bundle diameter would influence percent activation and lead distance values, but the general relationships and trends established in this study would not substantially differ from what we have reported.

The use of a generic, straight, fiber bundle target and surrounding avoidance region also influenced the calculation of overall robustness performance. Modeling the exact geometry of the target and avoidance regions for each anatomical target will produce different results, possibly shifting the performance differences we observed between each lead design. All tested lead locations and approaches were equally weighted when calculating the overall robustness of performance. Similarly, all off-target stimulation penalties were equally weighted in the optimization algorithm, regardless of position and distance from the target region. This initial study is not meant to mimic any one clinical DBS target but instead to establish a framework to understand the possible variations in target activations due to lead location, lead design, and multi-lead configurations.

## 5. Conclusions

The framework developed in this study can facilitate the development of novel lead designs to determine optimal electrode spacing and geometries with simulations before the leads are manufactured. The concept of robustness can also be applied directly to clinical DBS use cases to determine which available lead design would perform best, given patient-specific constraints such as target location and probable lead trajectories. For cohort-level analysis of DBS outcomes, robustness analysis can be used to quantify the variability in target activation based upon each patient’s lead location and stimulation settings. These data would provide knowledge about how much outcome variability can be attributed to lead location when investigating the efficacy of DBS as a therapeutic intervention.
